# Open label phase II trial of single, ascending doses of MRA in Caucasian children with severe systemic juvenile idiopathic arthritis: proof of principle of the efficacy of IL-6 receptor blockade in this type of arthritis and demonstration of prolonged clinical improvement

**DOI:** 10.1186/ar1826

**Published:** 2005-09-15

**Authors:** Patricia Woo, Nicholas Wilkinson, Anne-Marie Prieur, Taunton Southwood, Valentina Leone, Polly Livermore, Helena Wythe, David Thomson, Tadamitsu Kishimoto

**Affiliations:** 1 Great Ormond Street Hospital, NHS Trust, London, UK; 2 Hopital Necker Enfants Malades, Paris, France; 3 University of Birmingham and Birmingham Children's Hospital, NHS Trust, Birmingham, UK; 4 Chugai Pharma Europe Ltd, London, UK; 5 Laboratory of Immune Regulation, Graduate School of Frontier Biosciences, Osaka University, Osaka, Japan

## Abstract

Eighteen Caucasian (white, Middle East and Asian) children diagnosed by paediatric rheumatologists in the UK and France as having systemic juvenile idiopathic arthritis (sJIA) were enrolled in this open label, single dose trial. All patients had evidence of continued symptoms and disease activity for at least three months while receiving >0.2 mg/kg/day of prednisolone, or its equivalent, prior to recruitment. Twelve patients also received methotrexate (≤20 mg/m^2^/week). The patients were divided into three groups receiving 2, 4 or 8 mg/kg of MRA (tocilizumab) by intravenous infusion. No evidence of dose-limiting toxicity was observed and there were no dose-limiting safety issues. MRA appeared to be dramatically effective, with clinical and laboratory responses observed by 48 h post infusion, and these improvements continued well after serum MRA was undetectable. Eleven patients achieved the JIA definition of improvement (at least 3 of 6 core set criteria with a 30% improvement and no more than one worsened by 30%) and eight achieved ≥50% improvement. There were no observable differences with age. Clinical improvement in these children was observed for up to eight weeks, supporting the hypothesis that IL-6 is a key cytokine in the upregulation of genes crucial in the inflammation processes of sJIA, and the possibility of sequestration of MRA in the extra-vascular compartment needs to be considered.

## Introduction

Juvenile idiopathic arthritis (JIA) is a heterogeneous group of persistent arthritides of unknown origin occurring before 16 years of age [1]. One subset, systemic JIA (sJIA), is defined by the additional presence of debilitating fever, evanescent rash, hepatosplenomegaly, lymphadenopathy and serositis. Severe complications include osteoporosis, growth retardation, systemic amyloidosis and macrophage activation syndrome and are observed more frequently in patients with long-standing disease than in other JIA subsets. Patients with sJIA have a range of other prominent features, including marked elevation of erythrocyte sedimentation rate (ESR) and C-reactive protein (CRP), leucocytosis with high neutrophil counts and thrombocytosis [2]. Ferritin concentrations are high and correlate with systemic disease activity [2,3]. Anaemia is microcytic and characterised by a marked defect in iron supply for erythropoiesis [4]. Virtually all children with sJIA are negative for antinuclear antibodies and rheumatoid factor [2,4]. The mean duration of active disease is five to six years in Caucasians, with disabling polyarthritis becoming prominent in up to 50% of patients, while the systemic features usually regress within three to four years [5]. Twenty three percent of these patients have poor outcomes as adults [6].

The polyarthritis of sJIA is often extraordinarily resistant to treatment. Steroids are used to control systemic symptoms, but do not alter long-term prognosis and are associated with severe side effects such as osteoporosis and growth failure, to which patients with sJIA appear particularly susceptible. Methotrexate, effective in other forms of paediatric polyarthritis, appears less effective in sJIA [7]. Etanercept has been shown to have limited efficacy, with a possible increased risk of macrophage activation syndrome [8,9]. There is no strong evidence of definite improvements with the use of cyclosporine, azathioprine, or cyclophosphamide [2]. Chlorambucil has been used to treat amyloidosis with an improvement in survival by inducing remission [2,10]: but, as with cyclophosphamide, there are concerns over its long-term use due to side effects [10]. Pilot data on anakinra suggest blocking IL-1 can be effective [11,12], but further placebo controlled/comparative trials are necessary. Moreover, as anakinra is a daily subcutaneous injection, which is well known to cause some discomfort and pain, it may not be widely tolerated by children. There is, therefore, a need for a more effective treatment for sJIA, based on an understanding of the underlying pathophysiology of the disease process that may alleviate the systemic features of sJIA, as well as prevent progression of joint damage.

There is a strong body of evidence that IL-6 production is particularly high in sJIA and that this is genetically determined by a variant of the gene encoding IL-6 in a significant proportion of patients [13,14]. In addition to the increase in serum IL-6, it has been found that there is a significant increase in soluble IL-6 receptor (sIL-6R) concentrations [15]. The large quantities of serum IL-6 present in IL-6/sIL-6R complexes have been found to be of particular biological relevance *in vivo *[16]. IL-6 levels correlate with disease activity, fever pattern and platelet counts, indicating an important role for IL-6 in the pathogenesis of sJIA [17-19]. Components of the IL-6 signalling pathway are, therefore, rational targets for the treatment of sJIA.

MRA (tocilizumab) is a humanized anti-human IL-6 receptor antibody of kappa-IgG_1 _subclass, developed collaboratively by Osaka University and Chugai Pharmaceutical Company Ltd (Japan). MRA is humanized by the grafting of complementarity determining regions of a mouse anti-human IL-6 receptor monoclonal antibody onto human IgG_1 _by recombinant DNA technology. It has been shown to compete for both the membrane-bound and soluble forms of human IL-6 receptor, thus inhibiting the binding of IL-6 to its receptor and its pro-inflammatory activity [20]. As IL-6 is postulated to play a pivotal role in the pathogenesis and disease activity of sJIA, MRA is being developed in this indication. An anti-IL-6 receptor monoclonal antibody, such as MRA, may have an important role to play, not only in relieving the patient of the systemic features of the disease, but also in preventing the progression of joint damage and reducing extra-articular complications of the disease.

The primary objectives of this trial were to determine the safety and tolerability of single, ascending doses of MRA in children with sJIA and to determine the pharmacokinetic profile of a range of single intravenous doses of MRA in children with sJIA, as recommended by the Medicines and Healthcare products Regulatory Agency, UK. The secondary objectives were to evaluate the efficacy of the range of single intravenous doses and to explore the potential for an antigenic response.

## Materials and methods

Eighteen children (white, Middle East and Asian Caucasians), diagnosed with sJIA and having active disease for at least three months while receiving greater than 0.2 mg/kg/day of prednisolone or its equivalent, were enrolled from the UK and France. Diagnosis was defined using the International League of Associations for Rheumatology criteria [1]. Ethics committee approval and written informed consent were obtained. All patients had at least one active systemic feature, one active joint and the doses of nonsteroidal anti-inflammatory drugs and steroids were unchanged for at least two weeks before the infusion of MRA. Concurrent methotrexate therapy was allowed to a maximum of 20 mg/m^2 ^weekly, stabilized for at least four weeks prior to administration of the study medication. Patients receiving other disease-modifying anti-rheumatic drugs went through a washout period (at least five half-lives of the drug) before inclusion. Children with a history of macrophage activation syndrome were excluded.

Patients were divided equally within two age ranges (2 to 5 and 6 to 18 years) to receive 2, 4 or 8 mg/kg of MRA by intravenous infusion. A safety review was conducted before progression to the next dose range. Clinical assessments were carried out before infusion, at 48 h post-infusion and weekly thereafter. The duration of follow up for the 2, 4 and 8 mg/kg groups was 4, 6 and 8 weeks, respectively. Clinical assessment was performed using the JIA core set criteria (parent global visual analogue scale (VAS), physician's global VAS, number of active joints, number of joints with limited range, the childhood health assessment questionnaire (CHAQ) and ESR) and the definition of improvement as defined by international consensus (3 or more core set criteria improved by 30% and no more than 1 core set criterion worsened by 30%) [21]. Improvements of 50% and 70% were defined as above, substituting 30 with 50 and 70, respectively. Systemic features were recorded, as previously described [7]. The Systemic Feature Score included an assessment of fever, rash, cervical lymphadenopathy, axillary lymphadenopathy, inguinal lymphadenopathy, hepatomegaly, splenomegaly and clinical evidence of serositis (pericarditis, pleuritis or peritonitis). In addition to any occurrence of fever at the assessment visit, the parents/caregivers were asked about the presence of fever in the 24 h period preceding the assessment visit as part of the Systemic Feature Score. The overall Systemic Feature Score could range from 0 to 8 and is the sum of the eight individual features, which were scored as either absent (0 points) or present (1 point).

Laboratory measurements included full blood count, liver (gamma-glutamyl transpeptodase (GGT), aspartate aminotransferace (AST), alanine aminotransferase (ALT), bilirubin) and kidney (urea, serum creatinine, albumin) function tests, serum CRP and ESR. These were performed in the accredited routine laboratories of the participating hospitals. Levels of serum MRA, IL-6, sIL-6R and anti-MRA antibodies were screened during the follow up period by contract laboratories of the sponsor. The sponsor of the trial was Chugai Pharma Europe Ltd. Due to the exploratory nature of the study, no formal sample size calculations were performed. The sample size was based on clinical judgment and practical considerations. No formal statistical analysis of any data was performed.

## Results

### Safety data

The baseline demographic data for the 18 patients enrolled is shown in Table 1. Fifteen patients reported 59 treatment-emergent adverse events and the majority were mild. The majority of events reported were classified as gastrointestinal disorders, abnormal investigations (including increases in hepatic enzymes), respiratory disorders and infection/infestations. Four patients reported nine drug-related adverse events. No serious bacterial infections were documented. One patient developed urticaria within 4 h of the end of the 2 mg/kg MRA infusion, lasting two weeks. Transient rises of ALT occurred in three patients who also received methotrexate, but all had above normal values of GGT and/or AST, lactic dehydrogenase (LDH), and ALT at screening, but were below the levels stated in the exclusion criteria. All but three patients had at least one low lymphocyte value, generally at one and two weeks after infusion; however, eight patients (44%) had lymphocyte counts below the local laboratories' normal ranges, (but above the level stated in the exclusion criteria) before infusion of MRA.

There were no withdrawals due to adverse events. Any adverse events were reported up to the end of the follow up period. Five serious adverse events were reported in four patients: chicken pox in one patient, contracted during an outbreak at his nursery; transient pancytopenia in one patient at week seven; disease flares requiring hospitalisation occurred in two patients after three and six weeks (one of these reported a second adverse event; a herpes simplex mouth ulcer that had previously occurred during flares of disease). There were two flares of sJIA (in patients that received 4 and 8 mg/kg MRA) that were considered to be possibly drug-related; one developed at two and the other at six weeks post-infusion.

### Efficacy data

Fifteen patients were included in the efficacy analysis (2 mg/kg (n = 4), 4 mg/kg (n = 6), 8 mg/kg (n = 5)); three patients were excluded due to protocol violations. At baseline, the systemic feature scores were generally comparable across the three treatment groups (Table 2 shows the baseline values for all 18 patients). There was considerable variability in baseline values for the physician's global VAS, total number of active joints, total number of joints with limitation of movement and ESR, with the 8 mg/kg treatment group appearing to have a less active disease state at baseline when assessed according to these parameters.

**Figure 1:**
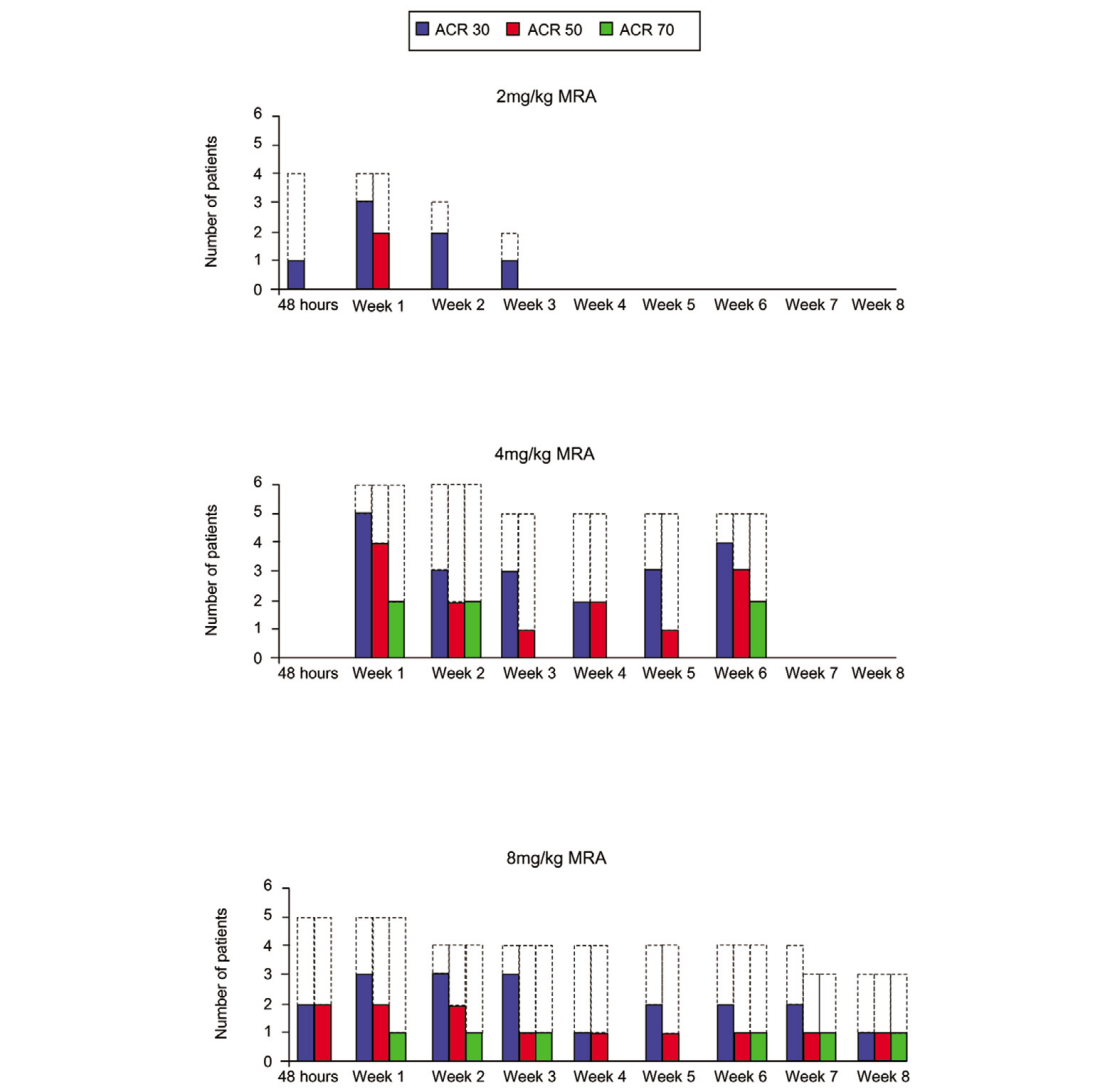
Cumulative number of patients improving from baseline in juvenile idiopathic arthritis core outcome variables (dotted lines show non-improvers); ACR, American College of Rheumatology.

A marked clinical response was noted at 48 h in all patients and in all three dose groups. Clinical improvement, as defined by Giannini *et al*. [21] (30% in at least 3 core sets and no more than one worsened by 30%), was achieved at week one in 11 patients. The clinical response in the 4 mg/kg group (n = 6) was the most dramatic, with four and two patients achieving 50% and 70% improvements, respectively, at week 1 (Fig. 1). The effect appeared to be more prolonged in the 4 and 8 mg/kg groups: four and three patients achieved 50% and 70% improvements, respectively, at week six. There was a trend towards a reduction in the mean number of joints with active disease/limited movement, although these parameters showed less of an improvement compared to the other core outcome variables. Moreover, parents reported dramatic changes in their child in terms of mobility and well-being (as measured by Parent's/Caregiver's or Patient's VAS of overall well-being (0 mm = very well, 100 mm = very poor). Age did not affect this response. The Total Systemic feature score also decreased within one week post infusion in all dose groups.

**Figure 2:**
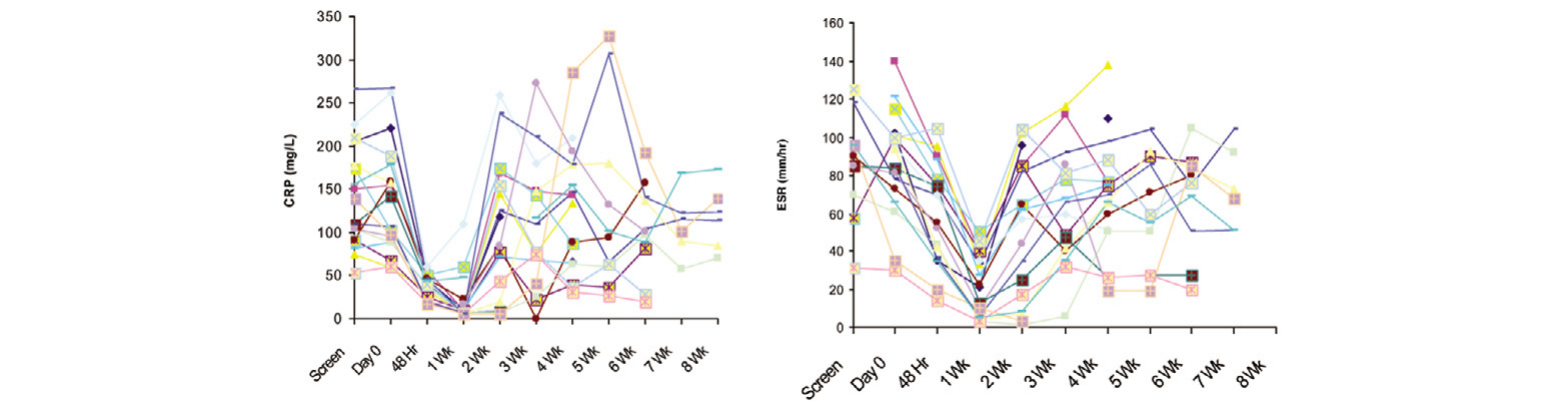
Serum C-reactive protein (CRP; mg/l) and erythrocyte sedimentation rate (ESR) of individual patients for all dose groups from day 0, pre-infusion, until the end of the follow-up period for each dose group.

**Figure 3:**
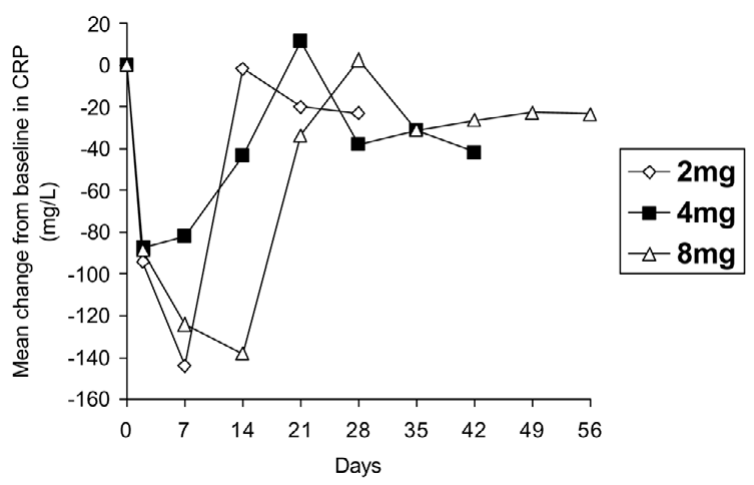
Mean changes from baseline (day 0, pre-infusion) in C-reactive protein (CRP; mg/L).

Clinical improvement was paralleled by a decrease in CRP levels (Fig. 2). There was a transient increase of mean CRP after weeks one or two, but mean levels were lower than baseline for the remainder of the follow up period (Fig. 3). The same profile was observed for ESR, but with a small time delay in response, the maximum decrease being observed at week one. There were also improvements in the haemoglobin, total white counts and serum albumin, with the peak improvements at one week after infusion. Interestingly, only three children required intervention with rescue therapy, such as prednisolone, even though CRP values tended to return to baseline. The treatment was not modified during the follow up period, except for two patients who required intravenous methylprednisolone at week six (MRA 4 mg/kg, and 8 mg/kg) and one requiring an increase in oral steroids from 5 to 10 mg 24 h after infusion (MRA 2 mg/kg). The clinical and biological improvements were maintained after MRA was undetectable in the serum.

Serum IL-6 and sIL-6R increased for two to four weeks after dosing, with both the magnitude and duration of this effect being dose-related. Following this initial increase, IL-6 levels were observed to decrease for the 4 and 8 mg/kg dose groups.

## Discussion

Single dosing with MRA in these 18 European Caucasian children does not appear to cause any drug related serious infections during the infusion and follow-up period. A report of multiple dosing in a group of Japanese children with a similar disease did not reveal serious infections as a significant side effect over one year [22].

An increased incidence of infection, including serious life threatening infections, has been observed with other monoclonal antibodies active against components of the immune system. In this study, infections were generally mild in intensity and the incidence of infections did not increase at the higher dose levels. Only the herpes simplex mouth ulcer during a disease flare three weeks post infusion was considered to be possibly related to MRA, although herpes simplex mouth ulcer has been reported previously during a disease flare of this patient. Of the nine infections reported, six were associated with a lymphocyte count below the lower limit of normal; however, lymphopenia was present in the majority of patients following dosing and nine patients with low lymphocyte counts did not have a corresponding infection. Further studies will need to address the longer term safety of MRA in a larger patient population, as this pilot study was to prove the safety of the therapy in a single dose setting.

MRA appears to be dramatically effective clinically in children with sJIA following a single infusion. Eleven patients achieved a clinical improvement – a 30% improvement in the composite score for disease activity in JIA (as defined by Giannini *et al*. [21]) – with maximum effect seen at one week for all the dose ranges. Eight and three patients, respectively, achieved 50% and 70% improvements in the composite score of disease activity mostly in the higher dose groups. The maximum duration of benefit was within four to eight weeks after dosing with all efficacy endpoints. The serum levels of the biological markers of the acute phase response (CRP and ESR) also showed dramatic decrease from baseline; many decreased 10-fold and, in a few patients, the serum CRP normalised. The prolonged effect of a single dose supports the hypothesis that IL-6 is a key cytokine in the upregulation of genes crucial in the inflammation processes of sJIA. This effect is in sharp contrast to the experience with single infusions of 30 mg/kg of intravenous methyl prednisolone that, given previously in these children, all resulted in transient and smaller clinical and laboratory improvements, lasting a maximum of 48 h before return to baseline. Response to MRA also compares well with anti-tumour necrosis factor therapy. Two infusions of 10 mg/kg of infliximab one week apart in a 16 year old sJIA patient led to some beneficial effect on the fever for only five days and no change in the joint symptoms [23]. Subsequent surveys of response rates to anti-tumour necrosis factor therapy have also shown that, at the most, there is significant improvement in only 30% [8,9]. Blockade of IL-6 signalling within inflammatory and immune cells could lead to secondary changes that may account for the prolonged well-being experienced by many patients in this small open trial. Another possible explanation of this prolonged clinical improvement in symptoms is that MRA may remain longer in the extravascular compartment; this possibility will need to be explored. Further controlled studies will need to be done, with multiple dosing, to assess the short and longer term efficacy and safety of MRA.

A recent report by Yokota *et al*. [22] using clinical and laboratory parameters similar to this study showed a similar and marked response in Japanese patients with sJIA to multiple dosing with MRA. The disease known as sJIA in Japan is very similar to that reported in Caucasians, but not enough is known about the phenotypes and genotypes to make a direct extrapolation of drug response. So this study provides further evidence in Caucasians that IL-6 is a key cytokine in this disease and blockade of its signalling by a monoclonal antibody produces dramatic and prolonged clinical and laboratory improvements.

## Conclusion

MRA given as a single intravenous infusion to severe sJIA patients was tolerated over the 2 to 8 mg/kg dose range studied. There was no evidence of dose-limiting toxicity and no dose-limiting safety issues were observed at any of the dose levels administered during the study.

Evidence of efficacy was observed at all dose levels studied, both in clinical and biological markers. The efficacy response appeared to be more limited at the lowest dose level (2 mg/kg). The overall clinical response appeared to continue after MRA was undetectable in serum and gradually decreased over a four to six week period following the infusion of MRA. These promising results with MRA in sJIA, a disease with an unmet medical need, will be further developed in phase III trials.

## Abbreviations

ACR = American College of Rheumatology; ALT = alanine aminotransferase; AST = aspartate aminotransferace; CHAQ = childhood health assessment questionnaire; CRP = C-reactive protein; ESR = erythrocyte sedimentation rate; GGT = gamma-glutamyl transpeptodase; IL = interleukin; JIA = juvenile idiopathic arthritis; LDH = lactic dehydrogenase; sIL-6R = soluble IL-6 receptor; sJIA = systemic juvenile idiopathic arthritis; VAS = visual analogue scale.

## Competing interests

The trial was sponsored by Chugai Pharma Europe Ltd. The authors would like to declare the following possible competing interests. NW, PL, HW and VL received funding from Chugai towards the cost of attending the PReS Scientific Meeting in Stresa during 2003. DT is a full-time employee of Chugai Pharma Europe Ltd.

## Authors' contributions

All authors contributed to and approved the final draft of this manuscript. PW had significant input into the study design; ethics committee submissions and protocol amendments; supervised the conduct of the study on patients at The Great Ormond Street Hospital, London; followed 11 patients that were entered; was the chief investigator of the study; reviewed the results and drafted and finalized the submitted manuscript. NW recruited, assessed and managed the ongoing care of patients; contributed to study amendments; was involved in data analysis; presented early outcome data to an international conference. A-MP entered and followed six patients through the study; participated in the discussion of the results. TS participated in the data gathering and verification; discussion of the data; drafting of the manuscript. VL participated in the patient selection process with TS; medical management and monitoring including dosing and follow-up visits; data collection and processing. PL participated in the patient selection process with PW and NW; medical management and monitoring including MRA dosing and follow-up visits; data collection and processing. HW participated in the data collection and processing of this study. DT participated in the supervision of the clinical and data management aspects of this study as Medical Director at Chugai Pharma Europe Ltd. TK discovered IL-6; developed anti-IL-6 receptor therapies for Castleman's disease and rheumatoid arthritis; collaborated with PW to obtain MRA for a trial in Caucasian children with sJIA.

## Figures and Tables

**Table 1:** Patient demographics for the safety population

	MRA		
	2 mg/kg (n = 6)	4 mg/kg (n = 6)	8 mg/kg (n = 6)

Age (years)			
Mean ± SD	6.3 ± 3.33	7.7 ± 5.13	4.8 ± 1.83
Median	6.5	6.5	5.0
Range	3, 10	3, 17	2, 7
			
Gender			
Male (n)	3	6	1
Female (n)	3	0	5
			
Race			
Caucasian (n)	6	6	6
			
Weight (kg)			
Mean ± SD	23.47 ± 10.954	27.45 ± 14.479	19.63 ± 4.286
Median	17.95	24.05	19.90
Range	13.8, 38.0	16.2, 55.5	14.6, 23.6
			
Height (cm)			
Mean ± SD	108.3 ± 14.07	117.5 ± 23.84	108 ± 12.92
Median	105.5	111.5	102.0
Range	90, 126	95, 159	94, 127

SD, standard deviation.

**Table 2:** Baseline characteristics of sJIA for the full analysis population

	MRA			
	2 mg/kg (n = 4)	4 mg/kg (n = 6)	8 mg/kg (n = 5)	Overall (n = 15)

VAS of disease activity^a ^(mm)				
Mean ± SD	60.3 ± 29.94	50.5 ± 22.10	35.3 ± 30.09	48.9 ± 26.49
Range	20, 92	17, 74	11, 73^f^	11, 92^g^
VAS of overall well-being^a ^(mm)				
Mean ± SD	42.8 ± 29.28	56.5 ± 34.85	59.2 ± 10.57	53.7 ± 26.42
Range	5,76	20, 100	50, 75	5, 100
Total number of active joints^b^				
Mean ± SD	27.8 ± 9.54	23.2 ± 13.01	12.4 ± 13.15	20.8 ± 13.07
Range	15, 36	4, 38	4, 35	4, 38
Total number of joints with limitation of movement^b^				
Mean ± SD	17.8 ± 10.28	15.2 ± 11.27	12.8 ± 13.10	15.1 ± 11.00
Range	7, 30	5, 35	5, 36	5, 36
ESR (mm/h)				
Mean ± SD	114.3 ± 20.45	78.0 ± 25.85	60.0 ± 18.13^f^	83.2 ± 30.07^g^
Range	95, 140	30, 100	35, 78	30, 140
CRP (mg/l)				
Mean ± SD	140.8 ± 90.08	119.0 ± 52.23	125.6 ± 39.48	NC
Range	59, 262	60, 189	89, 178	
Functional ability^c^				
Mean ± SD	0.7 ± 0.68	1.2 ± 0.89	2.3 ± 0.36	1.4 ± 0.91
Range	0.03, 1.5	0.13, 2.38	1.75, 2.75	0.03, 2.75
Total Systemic Feature Score^d^				
Mean ± SD	3.0 ± 1.41	3.2 ± 1.72	3.0 ± 2.45	3.1 ± 1.79
Range	2, 5	1, 6	0, 6	0, 6

^a^Measured on an increasing scale of 0 to 100 mm. ^b^Maximum of 81. ^c^CHAQ: maximum of 3. ^d^Maximum of 8. ^e^N = 2;individual patient's data presented. ^f^N = 4. ^g^N = 14. CHAQ, childhood health assessment questionnaire; CRP, C-reactive protein; ESR, erythrocyte sedimentation rate; NC, not calculated; SD, standard deviation; VAS, visual analogue scale.
